# Locally injected Mesenchymal Stem Cells optimize angiogenesis by regulating VEGF and CD31 expression in duodenal perforation

**DOI:** 10.1016/j.amsu.2022.104529

**Published:** 2022-09-02

**Authors:** Eko Setiawan, Bambang Purwanto, Brian Wasita, Agung Putra

**Affiliations:** a Doctorate Student of Medical Sciences, Faculty of Medicine, Universitas Sebelas Maret, Surakarta, Indonesia; b Department of Surgery, Faculty of Medicine, Universitas Islam Sultan Agung, Semarang, Indonesia; c Doctorate Program of Medical Sciences, Faculty of Medicine, Universitas Sebelas Maret, Surakarta, Indonesia; d Department of Internal Medicine, Faculty of Medicine, Universitas Sebelas Maret, Surakarta, Indonesia; e Department of Pathological Anatomy, Faculty of Medicine, Universitas Sebelas Maret, Surakarta, Indonesia; f Stem Cell and Cancer Research (SCCR) Laboratory, Faculty of Medicine, Universitas Islam Sultan Agung, Semarang, Indonesia; g Department of Pathological Anatomy, Faculty of Medicine, Universitas Islam Sultan Agung, Semarang, Indonesia; h Department of Postgraduate Biomedical Science, Faculty of Medicine, Universitas Islam Sultan Agung, Semarang, Indonesia

**Keywords:** Duodenal perforation, MSCs, Angiogenesis, CD31, VEGF

## Abstract

**Background:**

Duodenal perforation is considered as one of gastrointestinal emergency with high morbidity and mortality rate. The MSCs have the ability to improve wound healing by releasing several growth factors and anti-inflammatory cytokines to promote the angiogenesis process. This study aimed to investigate the role of MSCs in duodenal perforation wound healing.

**Methods:**

MSCs were isolated from rat umbilical cord and injected into duodenal wound site at doses of 1.5x10 [(Putra et al., 2018) 66 cells for T1 group and 3x10 [(Putra et al., 2018) 66 cells for T2 group. The control group was treated by local injection of normal saline. The VEGF levels were measured by Western blot, while CD31 expression was analyzed using immunohistochemistry staining. All examinations were assessed on days 3 and 7.

**Results:**

Results showed a significant increase in VEGF and CD31 expression on days 3 and 7 (p < 0,05). The VEGF level was significantly decreased on day 7 compared to day 3.

**Conclusion:**

The administration of MSCs improved the angiogenesis process in duodenal perforation by enhancing VEGF and CD31 expression.

## Highlights

• Duodenal perforation is considered as one of gastrointestinal emergency with high morbidity and mortality rate.
• The MSCs have the ability to improve wound healing by releasing several growth factors and anti-inflammatory cytokines to promote the angiogenesis process.
• This study aimed to investigate the role of MSCs in duodenal perforation wound healing.
• Results showed a significant increase in VEGF and CD31 expression on days 3 and 7.
• The administration of MSCs improved the angiogenesis process in duodenal perforation.

## 1 Introduction

Duodenal perforation has a high mortality rate. Although it has a low incidence rate, it can cause a more severe condition compared to gastric perforation as it contains gastric acid, bile, and pancreatic juice which can leak into the peritoneal cavity after perforation and leads to peritonitis and eventually sepsis [1,2]. It also has a high reperforation rate which can lead to more serious complications and death [3]. Therefore, a new therapeutic approach is needed to achieve optimum healing.

Mesenchymal Stem Cells (MSCs) have the capability to differentiate into multiple cells lineage and release various anti-inflammatory cytokines and growth factors to promote wound healing [[4], [5], [6], [7]]. Multiple studies had reported its ability to improve various organ injuries with promising results [[8], [9], [10], [11]].

The angiogenesis process plays a crucial role in duodenal perforation as newly formed blood vessels will provide essential nutrition and oxygen to the growing tissues [12,13]. Vascular Endothelial Growth Factor (VEGF) is the most vital angiogenic factor in wound angiogenesis through several mechanism [14,15]. Thus, MSCs have been known for its capability to secrete VEGF and induce surrounding tissues to secrete more VEGF [[16], [17], [18], [19]]. This study aimed to investigate the role of MSCs in duodenal perforation wound healing.

## 2 Materials and methods

### 2.1 MSCs isolation and culture

MSCs were isolated from pregnant wistar rat's umbilical cord. Samples were collected in a sterile culture dish with 0,9% NaCl. After washing with phosphate-buffered saline (PBS), the umbilical cords were separated from its attachment. The umbilical cords were mechanically minced and blood vessels were removed. Each sample was cultured in 25T flasks containing Dulbecco's Modified Eagle's Medium (DMEM), fungizon, penicillin/streptomycin, and 10% Fetal Bovine Serum (FBS) for 3 min. Flasks were incubated at 37 °C and 5% C and the medium was changed with fresh complete medium every three days. MSCs will emerge in approximately 14 days. After reaching 80% confluence, cells were detached using BDTM accutaseTM cell detachment solution (cat No. 561527).

### 2.2 Animal model

An animal study was conducted and experimental procedures were approved by the Institutional Review Board of the Ethics Committee of the Medical Department, Sultan Agung Islamic University, Semarang, Indonesia. 24 male wistar rats weighing between 250 and 300 g were kept in polypropylene cages in a standard room maintained at 23–35 °C with 12 light-dark cycle and 40–70% humidity. The animals had free access to AIN 76A standard food and water. Experiments were carried out after 3 days of acclimatization.

After fasting for 12h, the rats were anesthetized with intraperitoneal ketamine (80 mg per kg body weight) and the duodenum was exposed along the midline abdominal incision. A 10-mm longitudinal incision was made at the first part of the duodenum. Afterward, the duodenal perforation was disinfected and closed by a 6/0 polypropylene (Ethicon) non-absorbable suture at 2-mm intervals.

### 2.3 MSCs administration

Rats were divided randomly into four groups comprising 6 animals in each group. Sham group received no treatment and intervention, only sutured on incision's closure. The control group received a local injection of 300 μL NaCl. The MSCs group received a local injection of umbilical cord MSCs at doses of 1.5x10 [6] cells (T1) and 3x10 [6] cells (T2).

### 2.4 Animal termination

Rats were terminated on days 3 and 7 using a cocktail (Ketamine 50 mg/kg, Xylazine 10 mg/kg dan Acepromazine 2 mg/kg). Duodenum were harvested using the en bloc technique and divided into 2 parts. The first parts were stored in a cryotube with no RNAase at −80 °C in RNA later for protein isolation, the later parts were fixated using neutral buffered formalin for a histopathology examination.

### 2.5 Immunostaining

Immunohistochemistry was performed to detect the expression of CD31. Slides were deparaffinized using xylene 1, 2, and 3 for 5 min, and then rehydrated with ethanol for 3 min. Antigen retrieval was performed by soaking the slides into citrate buffer and inserted into a decloaking chamber (Biogear) for 30 min at 900C. Blocking endogen peroxidases was performed using H2O2. Background Sniper (Starr Trek Universal-HRP Detection Kit) was used for background blocking, and then the slides were incubated for 20 min. Then, they were incubated in anti-CD31 antibody (Dako; Carpinteria, CA) in a humidity chamber at 40C overnight. Secondary antibody Trekkie Universal Link (Starr Trek Universal-HRP Detection Kit) was added and the slides were incubated at room temperature for 60 min. They were then incubated in TrekAvidin-HRP Label (Starr Trek Universal-HRP Detection Kit) at room temperature for 45 min. Mayer Hematoksilin (Bio-Optica Milano S.p.A) was used for counterstaining. The observation was then performed under light microscopes.

### 2.6 Western blotting

VEGF expressions was determined using western blotting. The lysate is made using extracted tissue which the sample preparation solution (RIPA, PMSF, NaF, and cocktail inhibitor protease) is added. Then the cells were centrifuged at 4 °C for 10 min. The obtained supernatants were used for protein quantification using 260 nm and 280 nm wavelength UV–Vis spectrophotometry consecutively to the cells lysate, then the volume were measured and loaded into each wells. Electrophoresis was performed by transferring each lysate into an Eppendorf and adding 10x loading buffer. The hardened gel was installed into each chamber and buffer SDS was added. The sample and prestained marker were then loaded into electrophoresis kit, and then transferred into PVDF buffer container. PVDF membrane were soaked with methanol and transferred into the container and runned for 1 h. The membrane then transferred into blocking buffer and incubated for 1 h Anti-VEGF antibody (ab9969, Abcam, Cambridge, UK) were added and incubated at 4 °C then washed 3 times then secondary antibody were added and incubated for 1 h. Visual detection were performed using luminograph (Atto).

### 2.7 Statistical analysis

Data were shown as the means ± standard deviation (SD). The calculations were performed using SPSS 20.0 (IBM Corp., Armonk, NY, USA). After applying normality and homogeneity tests for the studied variables, the statistical significance of independent quantitative variables was assessed by the ANOVA test, followed by Bonferroni posthoc analysis. A p-value of <0.05 was considered significant.

## 3 Results

### 3.1 Differentiation and characteristics of Mesenchymal Stem Cells

**Fig. 1:**
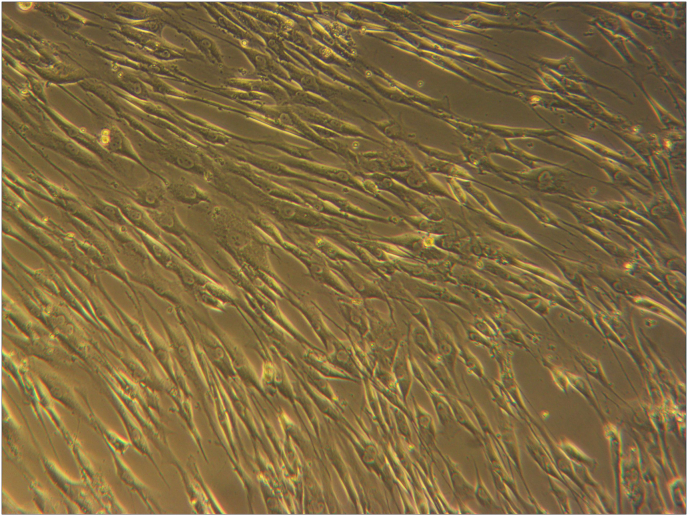
MSCs morphology with 90% confluency showed spindle-like cells (×200 magnification).

**Fig. 2:**
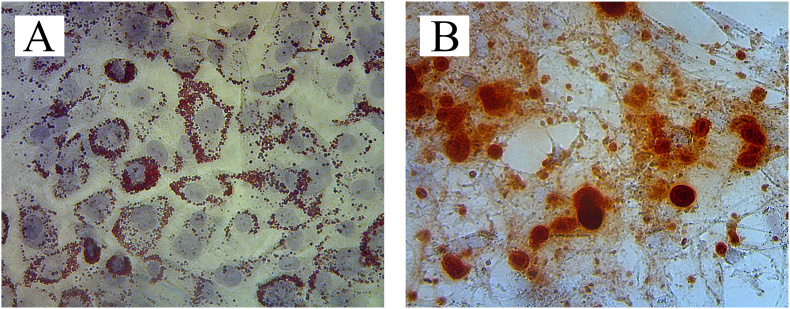
MSCs differentiation test using osteogenic and adipogenic culture media (×200 magnification. A: The brown staining indicates the presence of fat deposits in the culture that has been induced with adipogenic culture medium.; B: The red staining represents the result of post-cultured calcium deposits with osteogenic culture media. (For interpretation of the references to color in this figure legend, the reader is referred to the Web version of this article.)

**Fig. 3:**
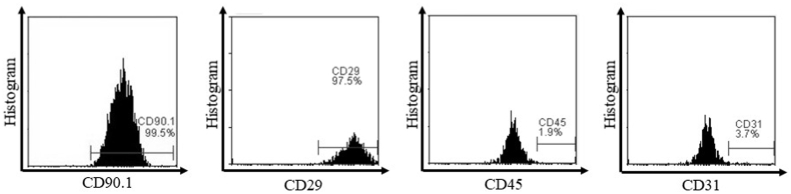
Flowcytometry analysis of CD90, CD29, CD45, and CD31 expression.

MSCs were isolated from rat umbilical cords which had been adhered to the culture flasks that were recognized as fibroblast-like cells morphologically (Fig. 1). It is induced to differentiate in terms of their phenotype and ability to give rise to osteogenic and adipogenic lineages (Fig. 2). Flow cytometric analysis revealed a positive expression for CD90 (99,8%) and CD29 (94,2%) while a negative expression for CD45 (1,8%) and CD31 (6,6%) (Fig. 3).

### 3.2 VEGF and CD31 expression

**Fig. 4:**
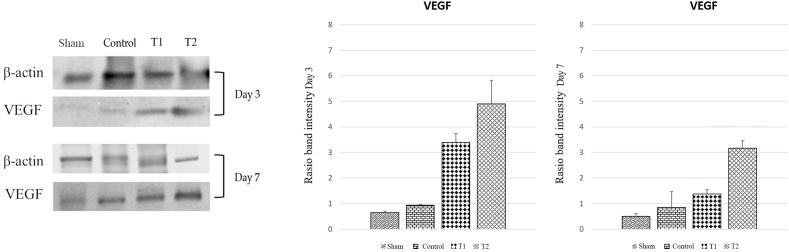
VEGF expression in duodenum in various study groups using Western blot. Thick band intensity showed an increase in VEGF expression with significant difference between control and treatment groups. There is also significant difference in VEGF level between T1 and T2 groups (p < 0.05).

**Fig. 5:**
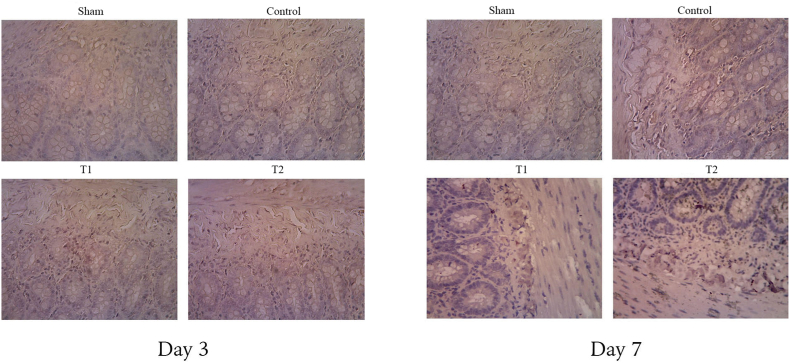
Expression of CD31 in duodenum tissue of various study groups using immunohistochemical methods (×400 magnification). CD31 expression is shown as brown colorization. (For interpretation of the references to color in this figure legend, the reader is referred to the Web version of this article.)

**Fig. 6:**
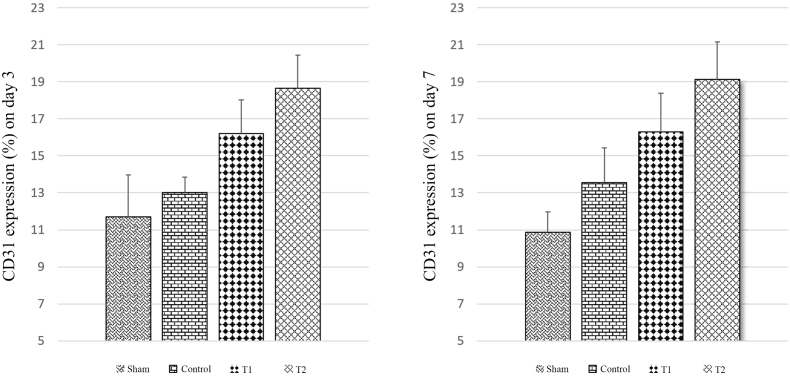
Quantification of CD31 staining showed significant difference between MSCs and control group. There is also significant difference in CD31 expresssion between T1 and T2 groups (p < 0.05).

VEGF has been shown to induce angiogenesis. There was a significant difference in VEGF levels between the four groups on both days 3 and 7. T1 demonstrated a higher VEGF level than the control group by 3,39 ± 0,21. However, the highest level of VEGF is found in T2 with 4,90 ± 0,51. It was also shown that the VEGF level was significantly decreased (T1 = 1,37 ± 0,10 and T2 = 3,16 ± 0,17) on day 7 compared to day 3 (Fig. 4). Similar results were shown in CD31 expression suggesting MSCs' ability to regulate angiogenesis (Fig. 5). On day 3, T1 demonstrated higher expression (16,20 ± 1,83) than control group (13,00 ± 0,84). T2 has the highest level of expression on day 3 (18,65 ± 1,79) and significantly increased on day 7 (19,14 ± 2,01). Meanwhile, there is no significant difference in CD31 expression in T1 between day 3 and 7 (see Fig. 6).

## 4 Discussion

The use of MSCs to treat gastrointestinal perforation has shown a promising treatment approach, especially in duodenal perforation wound healing. Umbilical cord MSCs propose the best stemness property among all other sources [20]. They play important role in wound healing by facilitating proliferation and angiogenesis process, producing extracellular matrix, and also suppressing the inflammation process [21]. Several important angiogenic growth factors produced by MSCs are Vascular Endothelial Growth Factor (VEGF), Transforming Growth Factor β (TGF-β), Fibroblast Growth Factor 2 (FGF-2), and Insulin Growth Factor 1 (IGF-1) [22].

MSCs have the ability to regulate angiogenesis as they produce many proangiogenic factors such as PDGF, EGF, FGF, TGF, PlGF, IGF, and the most potent one, VEGF. Furthermore, they help regulate cell surface protein involved in angiogenesis markers such as PECAM-1/CD31 [23,24]. These factors promote blood vessels growth which in turn deliver adequate nutrition and oxygen into the wound site and facilitate cell proliferation and tissue repair. To achieve optimal results, the MSCs need to be injected into the wound site as it has a longer half-time [11].

In this study, the significant increase in VEGF and CD31 expression on day 3 in the treatment groups suggests an increase in new blood vessels formation in the wound site. This in turn will deliver nutrients and oxygen to facilitate cell proliferation and tissue repair of the perforated duodenum. It also accelerates wound closure, improved granulation, and re-epithelization of the surrounding tissue [7]. This finding is supported by previous studies reporting MSCs' ability to improve angiogenesis in wound healing [5,8].

No study has been conducted to evaluate the potential benefit of MSCs in duodenal perforation, but a previous study on gastric perforation wound healing showed promising treatment. Liu et al. confirmed that local injection of MSCs in animal models decreased CD31 expression, suppressed IL-6, and increased TGF-β1 expression [25].

This study also shows a significant decrease in VEGF and CD31 expression on day 7 in the treatment groups compared to day 3, which suggests the ability of MSCs to regulate their expression. Although angiogenesis is essential in earlier stages of wound healing, excessive new blood vessels formation in later stages of wound healing is not needed, as it can cause the formation of scar tissue which leads to duodenal stenosis [26]. This finding aligns with previous studies reporting the formation of tissue fibrosis in the wound site triggered by the high level of VEGF in the remodeling phase of wound healing [27,28].

This study has limitations in which we did not investigate the potential side effect of umbilical cord MSCs administration. Hence, further studies with a larger sample size and more comprehensive follow-up are needed to examine and analyze the possible side effect of locally administered MSCs.

## 5 Conclusion

The administration of MSCs improved duodenal perforation wound healing by enhancing VEGF and CD31 expression at the angiogenesis process.

## Ethical approval

Ethical approval has been given from the Commission of Medical Research Bioethics, Faculty of Medicine, Sultan Agung Islamic University with reference number: No.150/V/2021/Komisi Bioetik

## Sources of funding

We report no involvement of any sponsor or funding body for this study.

## Author contribution

ES conceptualized the study, collected data and wrote the paper. BP conceived the theoritical framework, BW designed the study, criticized the manuscript, AP designed a laboratorium study and analyzed data. All authors read and approved the final manuscript.

## Availability of data and materials

The clinical and imaging data supporting the analysis and findings of this study will be available from the corresponding author upon reasonable request.

## Consent

Written informed consent are not required for this study.

## Guarantor

Eko Setiawan.

## Provenance and peer review

Not commissioned, externally peer reviewed.

## Declaration of competing interest

All authors have declared that they have no potential competing interests.
